# Responses of human colon and breast adenocarcinoma cell lines (LoVo, MCF7) and non-tumorigenic mammary epithelial cells (MCF-10A) to the acellular fraction of packed red blood cells in the presence and absence of cisplatin

**DOI:** 10.1371/journal.pone.0271193

**Published:** 2022-07-08

**Authors:** Kamila Czubak-Prowizor, Anna Macieja, Tomasz Poplawski, Halina Malgorzata Zbikowska

**Affiliations:** 1 Department of General Biochemistry, Faculty of Biology and Environmental Protection, University of Lodz, Lodz, Poland; 2 Department of Cytobiology and Proteomics, Medical University of Lodz, Lodz, Poland; 3 Department of Molecular Genetics, Faculty of Biology and Environmental Protection, University of Lodz, Lodz, Poland; 4 Department of Chemistry and Clinical Biochemistry, Medical University of Lodz, Lodz, Poland; Lobachevsky University, RUSSIAN FEDERATION

## Abstract

Perioperative blood transfusion in colorectal and some other cancer patients has been linked to the increased risk for recurrence, but a causal mechanism remains unclear. During the preparation and storage of packed red blood cells (PRBCs) bio-active substances accumulate in the acellular fraction (supernatant). Viability, proliferation, reactive oxygen species (ROS) levels, and DNA damage of colon (LoVo) and breast (MCF7) adenocarcinoma cells and non-tumorigenic MCF-10A cell line were determined in response to the supernatants of fresh and long-stored (day 42) PRBCs, leukoreduced (LR) or non-leukoreduced (NLR). The effect of supernatants on the cytotoxicity of cisplatin (cisPt) towards the cells was also examined. Supernatants, especially from a day 1 PRBCs, both LR and NLR, reduced the viability and inhibited proliferation of tumor cells (LoVo, MCF7), accompanying by the excessive ROS production, but these were not the case in MCF-10A. Moreover, supernatants had no effect on the cytotoxicity of cisPt against LoVo and MCF7 cells, while caused increased drug resistance in MCF-10A cells. The findings suggest the acellular fraction of PRBCs does not exhibit any pro-proliferative activity in the cancer cell lines studied. However, these are pioneering issues and require further research.

## Introduction

Anemia is a well-defined complication (hemoglobin (Hb) level < 11.0 g/dL; a drop below 6.5 g/dL is life-threatening) associated with cancer diseases. It has been recognized as a negative prognostic factor, who is directly related to the deterioration of the patient’s quality of life. Cancer-related anemia (CRA) has a multifactorial etiology and can result from a heavy blood loss during surgery, bone marrow infiltration by neoplastic cells (as in leukemia), chronic renal failure, and treatment (as a result of radiotherapy or chemotherapy) [1].

The standard treatment for CRA is transfusion of packed red blood cells (PRBCs). However, treatment with blood components is always associated with an increased risk of post-transfusion reactions, with transfusion-related immunomodulation (TRIM) a common consequence of PRBC transfusion [2]. A growing body of clinical evidence suggests that PRBC transfusion may lead to negative clinical outcomes (disease relapse, increased mortality, overall poor prognosis, metastasis) in patients with various cancers (e.g. esophageal, stomach, liver, ovarian cancer) [3, 4]. The transfusion-related decreased immune surveillance has been linked to cancer recurrence and progression [5]. An association between perioperative transfusion of allogeneic blood products and risk for recurrence has been shown in colorectal [3, 6, 7] and few other cancers [8], but the mechanisms of this phenomena have not been clarified yet. Due to the ambiguous literature reports and the small number of well-conducted randomized controlled trials, whether perioperative PRBC transfusion increases mortality and the risk of cancer recurrence in patients after surgical intervention is still debatable [9, 10].

During processing and storage, red blood cells undergo numerous biochemical and physiological changes with the concomitant accumulation of numerous bio-active substances mainly released from erythrocytes, but also residual contaminating leukocytes and platelets, into the acellular fraction of the PRBC unit. These include i.e. extracellular Hb, heme, iron, proteolytic enzymes, pro-inflammatory cytokines, chemokines, immunomodulating and vasoactive mediators, lipids, and microparticles (MPs) [11]. It was reported that some soluble factors present in PRBCs can directly stimulate tumor growth and spread [5]. Pre-storage leukoreduction reduces 3 log (99.9%) leukocyte numbers [12] which significantly decreases the accumulation of metabolites and cellular components into the PRBC unit [13].

Cisplatin, or cis-diamminedichloroplatinum (II), [PtCl₂(NH₃)₂] (cisPt) is a well-known cytostatic drug. Cisplatin and its derivatives have been clinically proven to combat different types of malignancies, including breast, testicular, ovarian, cervical, prostate, head and neck, bladder, lung, and many other cancers [14]. However, due to the drug resistance and numerous undesirable side effects, several combination therapies of cisPt with other drugs have been highly considered.

To the best of our knowledge, only a few earlier reports have evaluated the response of cancer cells to PRBCs [15–17]. Moreover, no previous study has addressed the effects of PBRCs on the cytotoxic effect of an anticancer drug. Therefore, the aim of the study was to evaluate the effect of the acellular fraction (supernatant) of the fresh (day 1) and stored (day 42) PRBCs (either pre-storage leukoreduced or non-leukoreduced) on the viability, proliferation, and levels of DNA damage and ROS in the colon (LoVo) and breast (MCF7) adenocarcinoma cell lines and in non-tumorigenic mammary epithelial cells (MCF-10A), in the absence and presence of cisPt.

## Materials and methods

### Chemicals

Cell Counting Kit-8 (CCK-8), cisplatin (cisPt), Cell Proliferation ELISA—BrdU colorimetric Kit (Roche), DAPI (4′,6-diamidino-2-phenylindole dihydrochloride), 2′,7′-dichlorofluorescin diacetate (H_2_DCF-DA), penicillin-streptomycin solution, normal melting-point agarose (NMP), Triton^™^ X-100, hydrogen peroxide, human recombinant insulin solution, and low melting-point agarose (LMP) were purchased from Sigma-Aldrich Chemicals (St. Louis, MO, USA). Dulbecco’s Modified Eagle Medium (DMEM) with 4.5 g/L glucose with L-glutamine, and MEGM^™^ Mammary Epithelial Cell Growth Medium BulletKit^™^ (MEBM^™^ Basal Medium and MEGM^™^ SingleQuots^™^ Supplements) were obtained from Lonza (Basel, Switzerland). Dulbecco’s Phosphate Buffered Saline without calcium and magnesium (DPBS), Hanks BSS (HBSS) without phenol red with calcium and magnesium were purchased from Biological Industries (Cromwell, CT, USA). Heat-inactivated fetal bovine serum (FBS) and trypsin-EDTA solution were from Biowest (Nuaillé, France). All other analytical grade and high-quality chemicals were obtained from local commercial suppliers, such as Chempur (Piekary Slaskie, Poland) or POCH S.A. (Gliwice, Poland).

### The packed red blood cells

Five SAGM-preserved PRBC transfusion units were purchased from the Regional Blood Donation and Blood Treatment Centre in Lodz (Poland**)**. Each of the SAGM-preserved non-leukoreduced (NLR; leukocytes <10^9^/unit) PRBCs, prepared in accordance with the standard procedures currently applied in blood banks, were divided into four equal aliquots by transferring them to the transfer bags in a closed system. Leukocytes from two transfer bags were removed during the gravity filtration process using a leukocyte depletion filter (BioR Flex, Fresenius Kabi AG, Bad Homburg, Germany) to obtain the pre-storage leukoreduced PRBCs (LR; leukocytes <10^6^/unit), the other two transfer bags were left unfiltered. All processes were carried out under sterile conditions in the Regional Blood Donation and Blood Treatment Centre (Lodz). The NLR and LR PRBCs had been stored, for 1 day or 42 days, at 4±2°C. The University of Lodz Research Ethics Committee approved the study (no. 26/KBBN-UŁ/I/2017) and waived the need for participant consent. The study did not include minors.

### Preparation of the supernatants from stored PRBCs

Supernatants from PRBCs, both NLR and LR, on day 1 (sNLR1 and sLR1, respectively) and day 42 of storage (sNLR42 and sLR42) were obtained in accordance with the method described by Westerman et al. [18]. Briefly, PRBC units were centrifuged at 2,000 g for 10 min at 4°C, the supernatants were removed and spun at 3,000 g for 10 min at 4°C. Next, supernatants were filtered through a syringe PES filter membrane with pore size 0.22 μm (TPP Techno Plastic Products AG, Switzerland), aliquoted, and stored at -70°C. All steps were carried out aseptically using sterile equipment.

### Cell culture and treatment

Human cancer cell lines: colon (LoVo; ATCC^®^ CCL-229^™^) and breast (MCF7; ATCC^®^ HTB-22^™^) adenocarcinomas, and human MCF-10A (ATCC^®^ CRL-10317^™^) non-tumorigenic mammary epithelial cell line, were obtained from the American Type Culture Collection (ATCC; Manassas, VA, USA). LoVo and MCF7 cell lines were grown in DMEM (MCF7 cells in the base medium supplemented with human recombinant insulin at the final concentration of 0.01 mg/mL). The complete growth medium for MCF-10A was MEBM^™^ Basal Medium supplemented with MEGM^™^ SingleQuots^™^ Supplements. Growth media (except for MCF-10A) were supplemented with 10% heat-inactivated FBS and 1% penicillin-streptomycin solution (10,000 units penicillin and 10 mg streptomycin/mL). Cells were grown in a humidified atmosphere with 5% CO_2_ at 37°C.

For each experiment, cells were seeded on 96-well tissue culture microplates and grown for 24 h in a complete culture medium supplemented with 5% by volume of PRBC supernatants (sNLR1, sLR1, sNLR42, sLR42) or with medium alone as a control, in the presence or absence of cisPt. The optimal concentrations of cisPt (25 μM for LoVo and MCF7, and 40 μM for MCF-10A) used in experiments were determined experimentally. First, IC_50_ was determined for each cell line, then a 2-fold lower cisPt concentration (½ IC_50_) was finally used in each experiment.

### Cell viability

To assess cytotoxicity after treatment with the supernatants the colorimetric assay, cell counting kit-8 (CCK-8), was conducted according to the manufacturer protocol. Briefly, cells (MCF7 and MCF-10A – 10,000 cells/well; LoVo– 15,000 cells/well) were grown in 96-well microplates for 24 h, then the supernatants and cisPt (where needed) were added to fresh culture medium in the plates. After 24 h, cells were washed twice with DPBS, then 100 μL of fresh media and CCK-8 solution (10 μL) were added to each well, followed by incubation for 3 h, at 37°C with 5% CO_2_. The absorbance at 450 nm was determined in the microplate reader SPECTROstar^®^ Nano (BMG LABTECH GmbH, Ortenberg, Germany). Cell viability was expressed as a percentage of the control (untreated) cells.

### Cell proliferation

Cell proliferation was quantified immunoenzymatically, using the bromodeoxyuridine (BrdU) proliferation assay, performed with a commercially available kit (BrdU colorimetric Kit, Roche) following the manufacturer’s instruction. Briefly, cells were cultured for 24 h in the presence of the 5% v/v of PRBC supernatants (with or without cisPt). After labeling with BrdU (at the final concentration of 10 μM, for 2 h at 37°C, 5% CO_2_), cells were fixed and the DNA was denatured by adding the FixDenat solution (incubation for 30 min at the ambient temperature). Next, cells were incubated with the anti-BrdU-HRP-conjugated antibody (90 min, ambient temperature). Cells were washed three times with washing buffer (PBS solution) and 100 μl of substrate solution for HRP was added (30 min, ambient temperature). The absorbance at 370 nm and 492 nm (reference wavelength) were measured in the microplate reader SPECTROstar^®^ Nano. Cell proliferation was expressed as a percentage of the untreated cells.

### Comet assay

The genotoxic effect of PRBC supernatants was assessed by the comet assay performed under alkaline conditions according to the procedure of Singh et al. [19] with some modifications [20]. Cells were incubated for 60 min at 37°C with 5 and 20% (v/v) of the supernatants. Cells suspended in fresh culture medium served as a negative control. Positive control was prepared by treating cells with 10 μM hydrogen peroxide. After incubation, supernatants were removed by centrifugation (180 g, 15 min, ambient temperature). Pellet of cells was suspended in 1.0% LMP agarose and spread onto microscope slides pre-coated with 0.5% NMP agarose. Next, the cells were lysed for 1 h, at 4°C in a lysis buffer (2.5 M NaCl, 100 mM EDTA, 1% Triton X-100, 10 mM Tris, pH 10). All remaining steps including electrophoresis, DAPI comet staining, and measuring were performed according to Czubatka et al. [21].

### Intracellular ROS

The intracellular ROS levels were measured using the H_2_DCF-DA fluorescent probe [22]. Briefly, cells were seeded onto a 96-well black/clear bottom microplate in the amount appropriate for the given cell line. After a 24-h culture, cells were incubated (30 min, 37°C, 5% CO_2_, in dark) with 10 μM H_2_DCF-DA dissolved in HBSS solution (140 mM NaCl, 5 mM KCl, 0.8 mM MgCl_2_, 1.8 mM CaCl_2_, 1 mM Na_2_HPO_4_, 10 mM HEPES and 1% glucose, pH 7.0). The fluorescent probe was then removed and the cells were washed twice with HBSS buffer. The PRBC supernatants and cisPt (where needed) to the final concentration of 10 μM (½ IC_50_) were added into wells. Cells treated with 10 μM H_2_O_2_ were used as positive controls, cells suspended in HBSS (untreated) as negative controls. After incubation (60 min, 37°C), fluorescence intensity was read at λ_ex_ = 485 nm excitation and λ_em_ = 538 nm emission (Fluoroskan^™^ Microplate Fluorometer; Thermo Scientific^™^).

### Statistical analysis

The results are presented as mean values ± SD. The normality of the results was analyzed using the Shapiro–Wilk test. Then, the non-parametric Levene’s test for homogeneity of variance was performed. Based on the Levene’s test (ANOVA) followed by the post-hoc Tukey test the differences between values were evaluated. All obtained data were analyzed using StatSoft Inc. “Statistica” v. 13.1. The value of p<0.05 are considered to be statistically significant. All presented figures were prepared using GraphPad Prism 5 Software.

## Results

**Fig 1:**
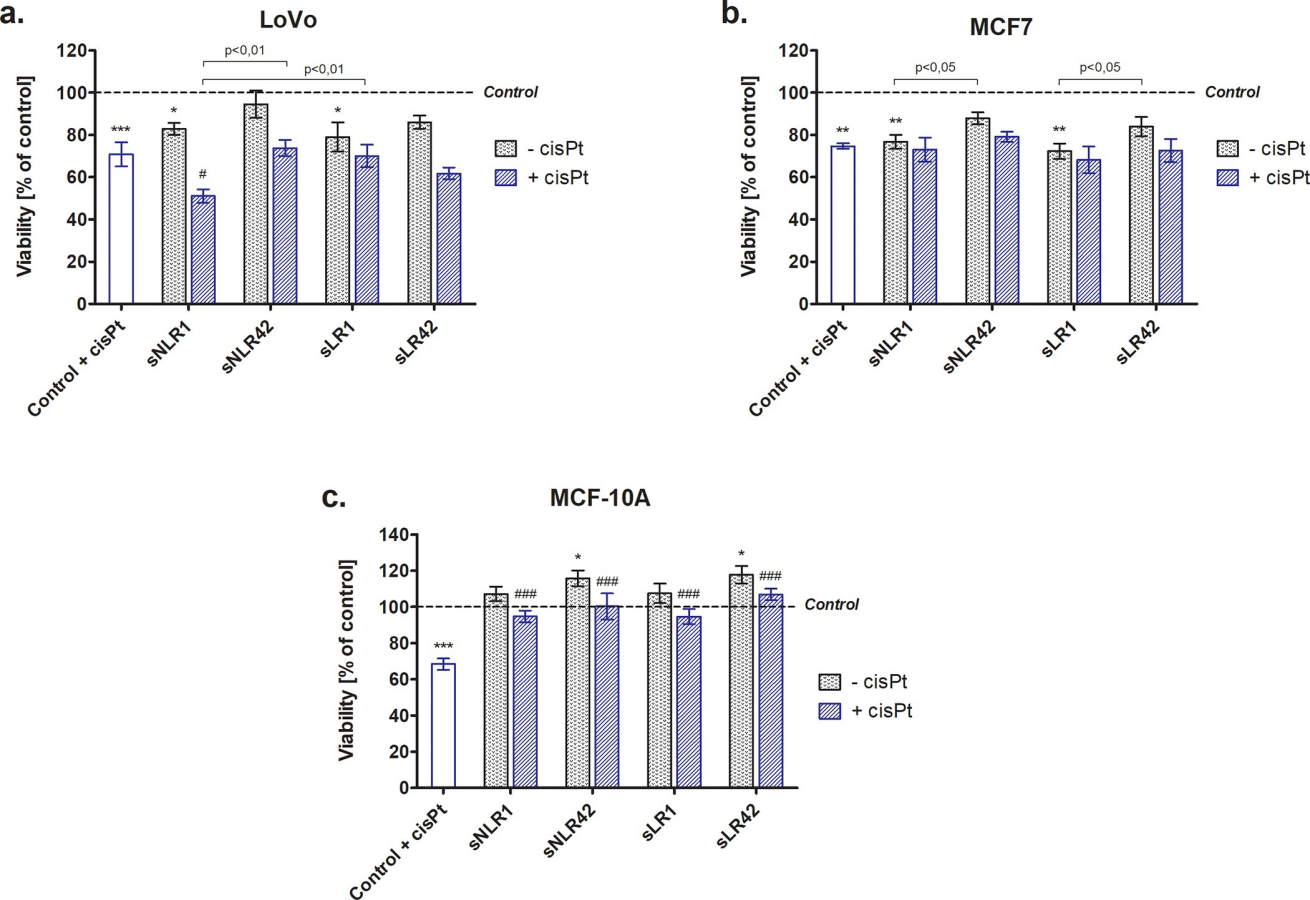
Viability of LoVo (panel a), MCF7 (panel b), and MCF-10A (panel c) cells after incubation (24 h) with the PRBC supernatants (5%) in the absence or presence of cisPt (panel a and b: 25 μM; panel c: 40 μM) evaluated using the CCK-8 assay. All data is presented as a percentage of control (untreated cells—assumed as 100%). Error bars denote ± SD, n = 15 (3 independent experiments, each performed with supernatants of PRBCs derived from five donors); *^/#^p<0.05; ***^/###^p<0.001 compared to the appropriate control, *p<0.05; **p<0.01; ***p<0.001 vs. control (untreated cells).

The viability of the human cell lines of solid tumors (LoVo, MCF7) and non-tumorigenic cells (MCF-10A) was assessed after 24-hour incubation with the PRBC supernatants (5% v/v per well) in the experimental system without the antitumor drug or in the presence of cisPt (½ IC_50_). Both the optimal concentration of supernatants and IC_50_ values for each line were determined experimentally. Cisplatin at these doses reduced the viability of LoVo, MCF-10A, and MCF7 cells by approximately 30% (p<0.001) and 25% (p<0.01), respectively (Fig 1A–1C).

Results presented in Fig 1A and 1B showed a significant reduction of the percentage of viable LoVo and MCF7 cells cultured in the presence of supernatants from the fresh (day 1 of storage) PRBCs, both sNLR and sLR, compared to untreated cells (control). A decline of LoVo viability was approximately 20% (p<0.05) and MCF7 in the range of 24–28% (p<0.01), in case of sNLR1 and sLR1, respectively. Experiments carried out with cisPt did not prove statistically significant changes in cancer cell survival, except for sNLR1 where a decrease in the viability of LoVo cells was noted (by 20%; p<0.05, compared to cells treated with cisPt alone) (Fig 1A and 1B). The supernatants from fresh PRBCs had no effect on the viability of the non-tumorigenic MCF-10A cell line, while their exposure to sNLR42 and sLR42 resulted in a higher percentage of viable cells (by approx. 17%; p<0.05, compared to control) (Fig 1C). The viability of MCF-10A cells cultured with supernatants and cisPt was found to reach the same level as in control cells.

**Fig 2:**
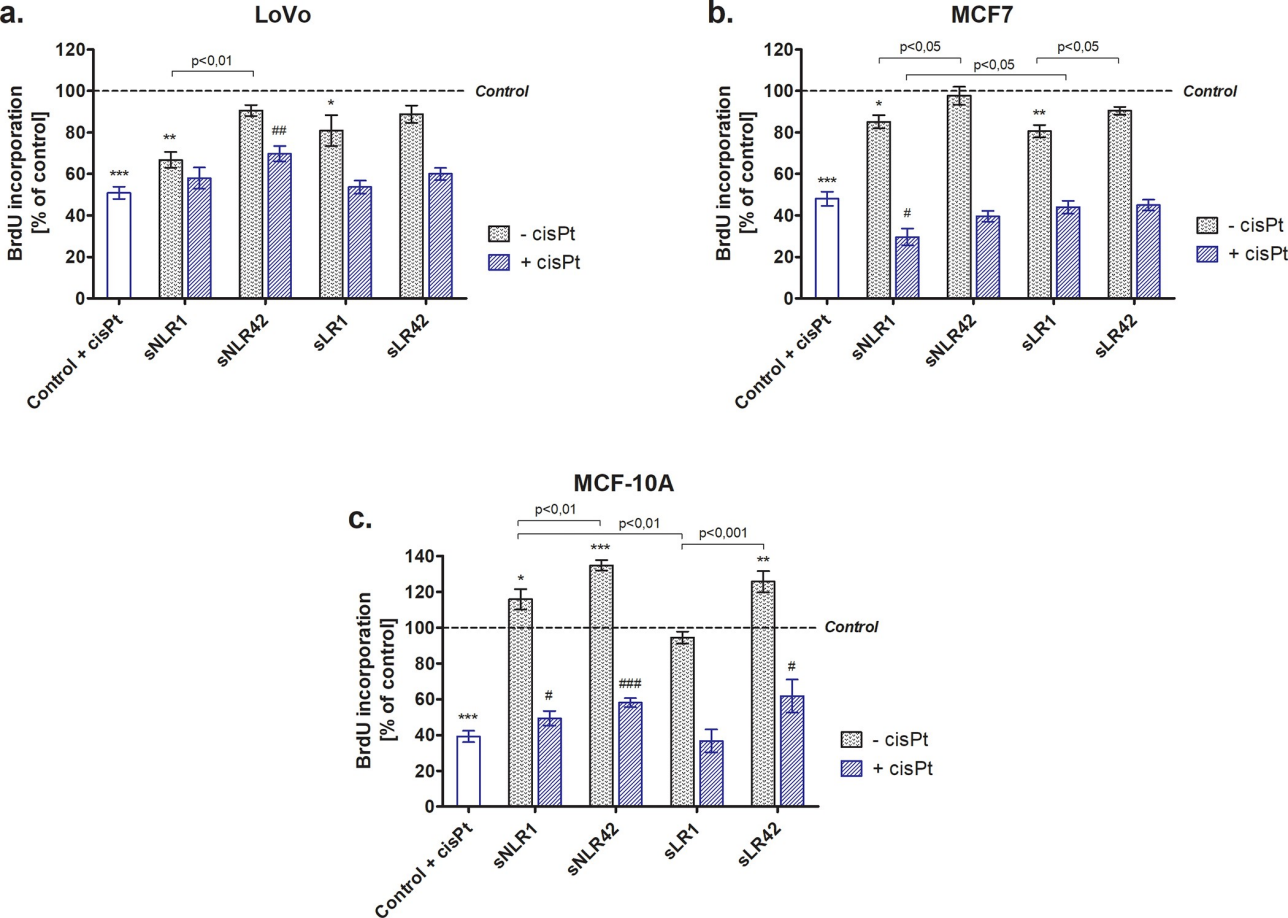
Proliferation of LoVo (panel a), MCF7 (panel b), and MCF-10A (panel c) cells, assessed by the BrdU incorporation during DNA synthesis, after incubation (24 h) with the PRBC supernatants (5%) in the absence or presence of cisPt (panel a and b: 25 μM; panel c: 40 μM). All data is presented as a percentage of control (untreated cells—assumed as 100%). Error bars denote ± SD, n = 15 (3 independent experiments, each performed with supernatants of PRBCs derived from five donors); *^/#^p<0.05; **^/##^p<0.01; ***^/###^p<0.001 vs. compared to the appropriate control.

Consistently with the results mentioned above (CCK-8 assay), the supernatants (sNLR1 and sLR1) significantly reduced the proliferation of LoVo cells (by above 30%; p<0.01) and MCF7 (by approx. 20%; p<0.01), assessed by the incorporation of BrdU (Fig 2A and 2B). MCF-10A cells grown in the presence of sNLR1, sNLR42 and sLR42 showed a better proliferation capacity than untreated cells (by 15%; p<0.05, 35%; p<0.001 and 25%; p<0.001, respectively) (Fig 2C). In the experimental system with cisPt the supernatants have generally not been found to change the proliferative capacity of cancer cells, except for sNLR42 (in LoVo) and sLR1 (in MCF7) where the increased or reduced BrdU incorporation were observed (p<0.01 or p<0.05), respectively. The increased proliferative capacity of MCF-10A cells has been found after exposure to the supernatants of long-stored PRBCs (by approximately 20%; p<0.001 and p<0.05 for sNLR42 and sLR42, respectively).

**Fig 3:**
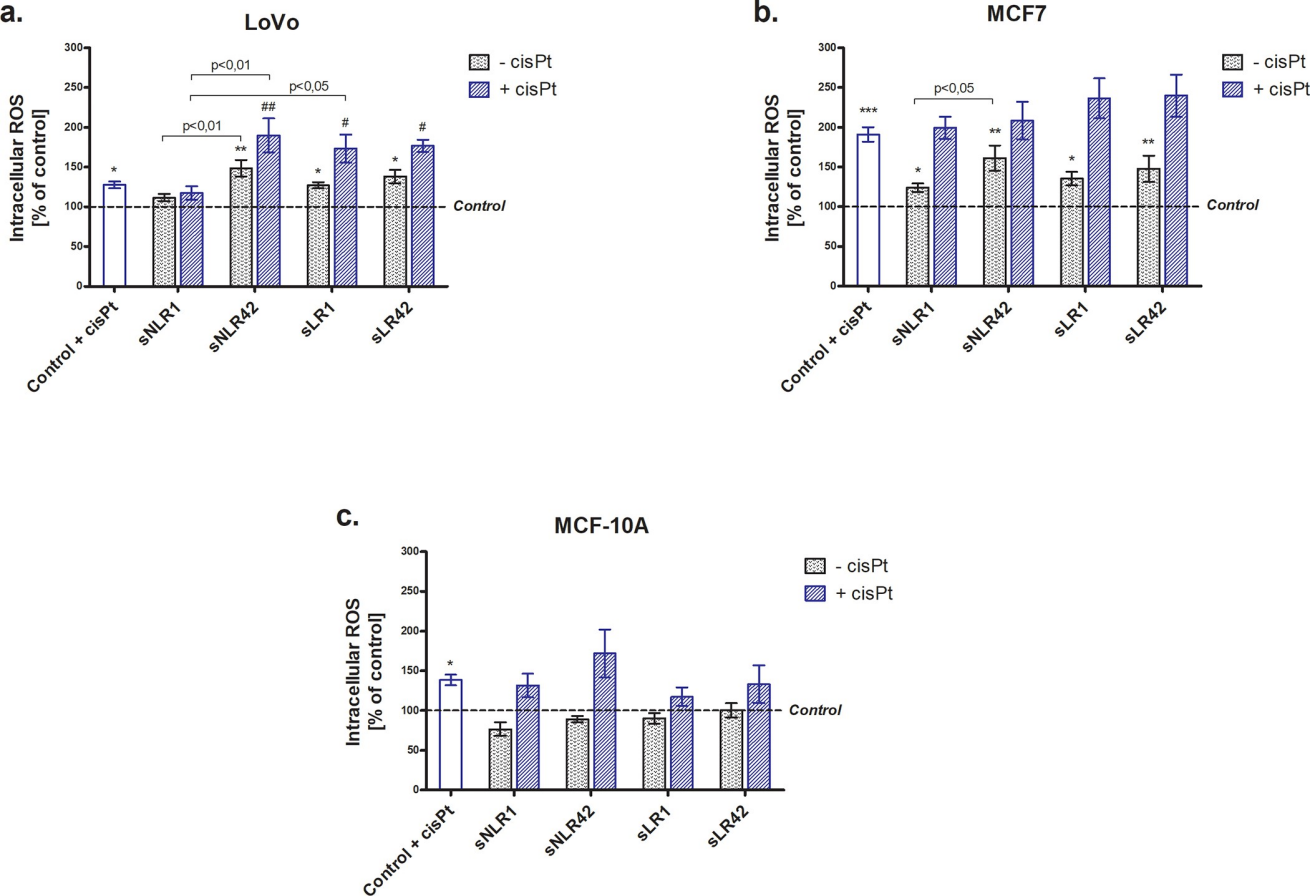
The intracellular ROS level in LoVo (panel a), MCF7 (panel b), and MCF-10A (panel c) after incubation (1 h at 37°C) with the PRBC supernatants (5%) in the absence or presence of cisPt (panel a and b: 25 μM; panel c: 40 μM). All data is presented as a percentage of control (untreated cells—assumed as 100%). Error bars denote ± SD, n = 25 (5 independent experiments, each performed with supernatants of PRBCs derived from five donors); *^/#^p<0.05; **^/##^p<0.01 compared to the appropriate control, ***p<0.001 vs. control (untreated cells).

The amount of intracellular ROS was determined using the H_2_DCF-DA fluorescent probe. As shown in Fig 3A, LoVo cells exposed to sNLR42, sLR1 and sLR42 displayed an increased level of ROS (by approx. 48% (p<0.01), 30% (p<0.05), and 38% (p<0.05), respectively). Similar, exposure of MCF7 cells to the supernatants resulted in the significantly increased ROS levels (compared to control cells by 24% (p<0.05), 61% (p<0.01), 35% (p<0.05) and 47% (p<0.01) for sNLR1, sNLR42, sLR1 and sLR42, respectively) (Fig 3B). It has also been noted that LoVo cells incubated with sNLR42, sLR1, and sLR42 and additionally with cisPt have generated much more ROS, compared to cells incubated with cisPt alone (sNLR42 by above 60% (p<0.01) and both supernatants of LR PRBCs by approx. 45% (p <0.05)) (Fig 3A). In contrast, the supernatants did not affect the ROS level in MCF-10A cell line (Fig 3C). Cisplatin-induced ROS production in all three cell lines, to the most extent in MCF7 (by approximately 90% that of control).

**Fig 4:**
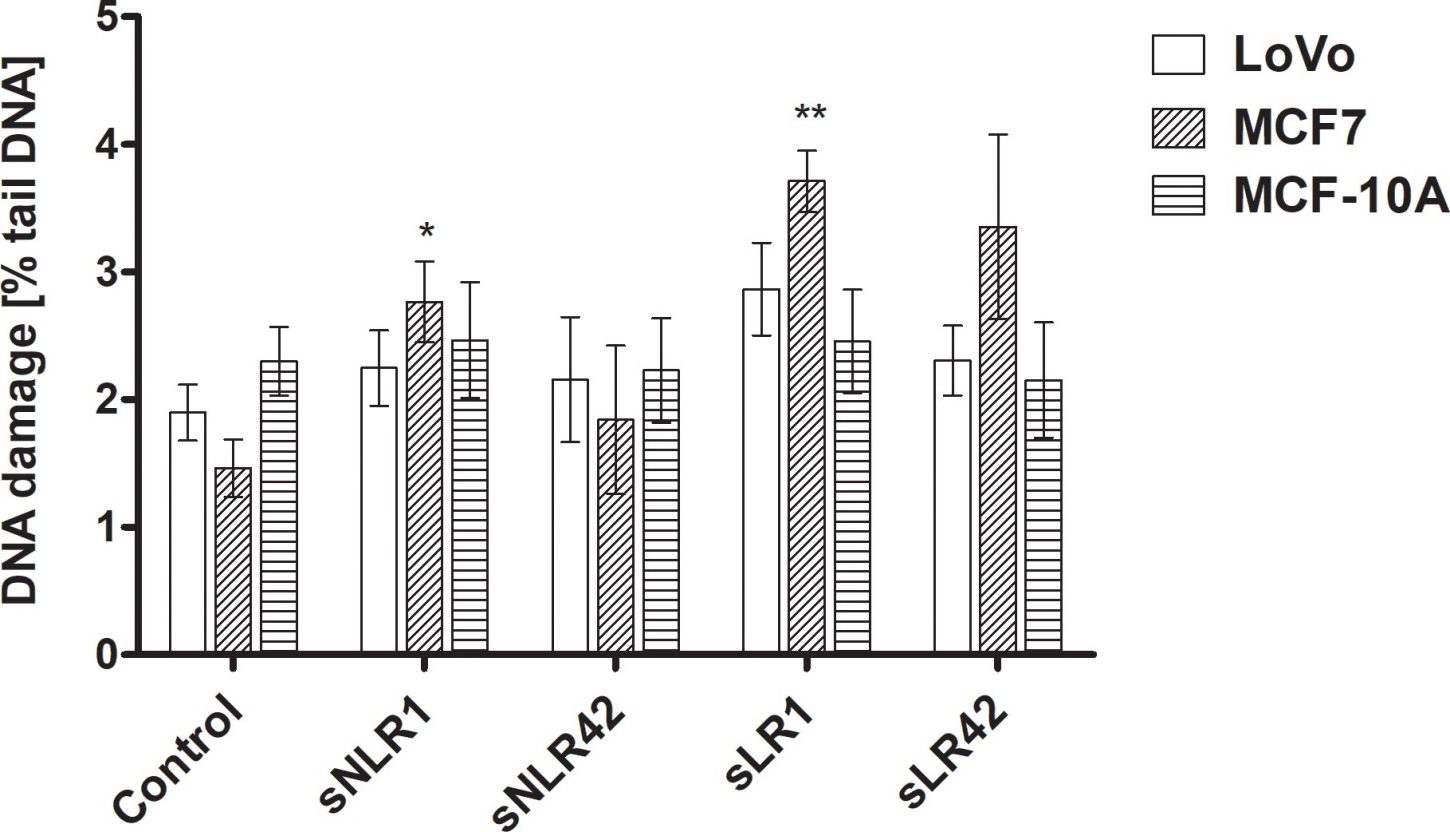
The DNA damage, measured as a percentage of tail DNA in the alkaline comet assay, in LoVo, MCF7, and MCF-10A cells pre-incubated for 1 h at 37°C with the PRBC supernatants (5%). All experiments included a negative (untreated cells) and positive (cells treated with 10 μM H_2_O_2_) control. Error bars denote ± SD, n = 15 (3 independent experiments, each performed with supernatants of PRBCs derived from five donors); *p<0.05; **p<0.01 compared to untreated cells.

It was shown that the level of DNA damage in MCF7 cells increased significantly after incubation with sNLR1 (by approx. 90%; p<0.05) and sLR1 (twice; p<0.01) compared to the control (Fig 4), although the observed level of DNA damage was very low (less than 5% of DNA in the "tail"). PRBC supernatants affected the level of DNA damage neither in LoVo cells nor in MCF-10A.

## Discussion

In cancer patients undergoing surgical resection of the tumor, a higher percentage of neoplastic disease metastases and recurrences was observed, and the risk of death related to perioperative PRBC transfusion [8, 23–26]. The causes of these events are believed to be the immunosuppressive effect of PRBCs, due to the impaired function of NK cells and cytotoxic T lymphocytes [9]. However, the mechanisms responsible for the increased risk of cancer progression or recurrence after PRBC transfusions are not fully understood, as well as the involvement of bioactive mediators and blood components, capable of activating angiogenesis and survival pathways in transformed cells, is disputable [2, 27]. Various biological mediators (e.g. cytokines, eicosanoids, growth factors) with the pro-tumoral activity have been identified in the acellular fraction of PRBC, suggesting that the accumulation of some of these substances during PRBC storage may have a tumor-promoting effect [12]. Therefore, we aimed to examine the effects of the supernatants of fresh and long-stored PRBCs (both NLR and LR) on the growth/proliferation of human colon (LoVo) and breast (MCF7) adenocarcinoma cells (examples of the solid tumors), and non-tumorigenic MCF-10A cells (as a reference). The goal was also to determine whether the supernatants can modify the cytotoxicity of cisPt towards these cells.

It was found that the supernatants (especially of 1-day PRBCs), both NLR and LR, significantly decreased the viability (Fig 1A and 1B) and inhibited proliferative capacity (Fig 2A and 2B) of LoVo and MCF7 cells. The stronger cytotoxic effect of supernatants isolated from fresh, compared to long-stored PRBCs clearly indicates that substances released as a consequence of the mechanical damage that blood cells suffer during initial manufacturing procedures (i.e. bio-active lipids and/or cytokines) rather than factors accumulating during storage, can cause such an effect. Recently, Karsten et al. reported that the storage-time dependent increase in cytokine levels results from their controlled release by intact RBCs but the mechanical disruption of these cells is also accompanied by the cytokine release whereby their levels may be high in 1-day PRBCs [28]. On the other hand, no effect of storage on the IL-6 concentration was demonstrated in the supernatants of unfiltered PRBCs [29]. Possibly, some antitumorigenic cytokines, i.e. interferons, IL-4, IL-6, IL-12, TNF-α, TGF-β [30], are responsible for the cytotoxic effect of the supernatants. These cytokines are known to be pleiotropic, and depending on the concentration or stage of the disease, they may have different properties. For example, their low concentrations may initiate and high ones inhibit the growth of neoplastic cells, which can also be the case under the experimental conditions.

Interestingly, supernatants did not show any suppressive activity against MCF-10A cell line (Figs 1C and 2C). Moreover, in the presence of the long-stored PRBC supernatants (sNLR42 and sLR42) MCF-10A cells have grown even better. The MCF-10A human breast epithelial cell line has been well characterized and extensively used as a model of pre-neoplastic (non-tumorigenic) cells. Immortalized MCF-10A cells need a basic growth medium with supplements and growth factors to grow properly. It is well known that during PRBC storage, numerous growth factors accumulate in supernatants, i.e. vascular endothelial growth factor (VEGF), epidermal growth factor (EGF), transforming growth factor β (TGF-β), insulin-like growth factor (IGF), basic fibroblast growth factor b-FGF [28, 31–33], which may explain the enhanced viability of these cells.

Our results remain inconsistent with the data published by Barnett et al. [15], who showed that supernatants of long-stored (unfiltered and filtered) PRBCs increased proliferation and migration of mouse pancreatic cancer cells (Pan02) after 24hours, and when administered intravenously contributed to pancreatic cancer progression in mice. The discrepancy between the findings may be related to the use of an entirely different cancer cell line, additionally of mouse origin. It is reasonable that substances released to PRBC supernatants may have diverse effects in different cancers. Moreover, Zhuang et al. reported the effect of platelet-derived growth factor (PDGF) and VEGF accumulating in PRBCs on the proliferation of human hepatocellular carcinoma cells (HepG2) [16]. The authors demonstrated that the supernatants of unfiltered PRBCs increased the proliferation of these cells [17]. Inconsistency in the results could have been due to not only different cell lines used, but also from other experimental setups. HepG2 cells were incubated with supernatants for 48 h, but importantly, the supernatant doses remain unknown, making comparison of results impossible (only Abstract available in English, results of these experiments were published in the original Chinese language). In our studies, the incubation time of cells with supernatants (24 hours) was selected due to the population doubling time. This time, however, may have been too short to properly assess the potential pro-proliferative properties of the supernatants. It has to be pointed out, that since we used the syringe filters with a pore size of 0.22 μm before freezing the supernatants used in the study, the participation of microparticles in the effect of the supernatants on cells can be largely excluded. Tayer et al. [34] also used 0.22 μm filters in their studies to remove MPs from samples.

Further, the level of intracellular ROS was found to be generally higher in LoVo and MCF7 cell lines cultured in the presence of supernatants, than in control cells, although it was not dependent on the PRBC storage duration and leukocyte counts (Fig 3A and 3B). At the same time, the supernatants did not cause oxidative stress in MCF-10A cells (no increase in ROS) (Fig 3C). Therefore, it cannot be excluded that one of the possible mechanisms responsible for the cytotoxic effect of the supernatants on cancer cells is the excessive ROS production. The observed effect can result from the presence of, i.e. Hb degradation products, bio-active lipids, and/or cytokines in PRBC supernatants. Free Hb, heme, and iron present in the supernatant are toxic to many cells [35], they can serve as Fenton reagent to initiate free radical reactions. High ROS levels have been suggested to promote anti-tumorigenic signaling by initiating oxidative stress-induced tumor cell death [36]. The excessive OS may induce cell cycle arrest, senescence, and cancer cell death either by the initiation of intrinsic apoptotic signaling in the mitochondria or by extrinsic apoptotic signaling through the death receptor pathways. Chronic OS and the accompanying inflammation are well-known factors influencing the progression of neoplastic disease [27]. Differential responses to supernatants between cancer cells (LoVo and MCF7) and non-neoplastic MCF-10A cell line may be due to the fact that neoplastic cells alone are characterized by high ROS levels, which result from, i.e. mitochondrial dysfunction and upregulated metabolic activities [37].

The elevated ROS in cells may contribute to the development of DNA damage (and other cellular biomolecules) and to the hyperactivation of ROS signaling pathways leading to cell death. Only in MCF7 cells, exposed to a day 1 supernatants, a slight increase in the level of DNA damage was observed (Fig 4B), although this was within the level of endogenous DNA damage. Single and double DNA strand breaks as well as alkali labile sites can be detected by the alkaline version of the comet assay. It is likely that if were not persistent the damage to DNA could have been repaired by the cellular DNA repair systems prior to testing or the applied dose of the supernatants was possibly not high enough to be genotoxic to cells. The results of the current study indicate that leukocyte counts did not significantly influence the response of cells to the supernatant. Pre-storage leukoreduction of PRBCs results in the removal of white blood cells by greater than 3 logs and a decrease in platelets by 4–5 logs, as well as a marked reduction in some but not all lipids [38]. In the fresh PRBC, the concentration of 12-HETE (involved in such processes as OS, inflammation, and neoplastic disease) does not differ between NLR and LR PRBCs [39].

We also showed that the supernatants had no effect on the cytotoxic properties of cisPt in the cancer cell lines (LoVo, MCF7). However, the survival of MCF-10A cells cultured in the presence of cisPt and supernatants was similar to that of untreated cells (Fig 1C) and the proliferative capacity was significantly increased (Fig 2C), suggesting supernatants to abolish/decrease the cytotoxicity of the drug. Cisplatin and its analogues have been used to treat a variety of solid tumors, both alone and in combination with other drugs [10]. It is generally considered as a cytotoxic drug that kills cancer cells by direct interaction with DNA through the formation of intra-strand bonds that inhibit replication, leading to cell cycle arrest and then apoptosis [2, 10]. A major obstacle to successful cisPt-based chemotherapy is the intrinsic and acquired resistance of tumor cells to this drug. Cisplatin-induced DNA damage activates various signaling pathways to prevent or promote cell death. The mechanism of the anti-tumor activity of cisPt is not fully understood. Only 1% of the cisPt present in a cell has been shown to bind to nuclear DNA. The remaining percentage reacts with membrane and cytoplasm components, which indicates that the drug may also exert a cytotoxic effect through mechanisms independent of nuclear DNA binding [40], e.g. by inducing the production of ROS [41]. Our findings suggest the involvement of ROS in the mechanism of the drug cytotoxic action on LoVo, MCF-10A, and especially MCF7 cells (Fig 3).

To sum up, under the experimental conditions used, PRBC supernatants reduced the viability and inhibited proliferation of tumor cells (LoVo, MCF7), but they do not show such an effect against the non-tumorigenic cell line (MCF-10A). The results indicate that one of the mechanisms responsible for the cytotoxic effect of the supernatants on cancer cells is excessive ROS production. A differentiated effect of the supernatant was also seen on the cytotoxicity of cisPt towards the cells tested. The substances present in the supernatant had no effect on the cytotoxicity of cisPt against LoVo and MCF7 cells, while they caused increased drug resistance in MCF-10A cells. Due to the innovativeness of the research, it is difficult to interpret the results and determine their clinical significance. It may be suggested that the acellular fraction of PRBCs does not exhibit any pro-proliferative activity in the cancer cell lines studied. However, this issue requires further research.

## Supporting information

S1 Table Viability of LoVo (panel a), MCF7 (panel b), and MCF-10A (panel c) cells after incubation (24 h) with the PRBC supernatants (5%) in the absence or presence of cisPt (panel a and b: 25 μM; panel c: 40 μM) evaluated using the CCK-8 assay.
(DOCX)

S2 Table Proliferation of LoVo (panel a), MCF7 (panel b), and MCF-10A (panel c) cells, assessed by the BrdU incorporation during DNA synthesis, after incubation (24 h) with the PRBC supernatants (5%) in the absence or presence of cisPt (panel a and b: 25 μM; panel c: 40 μM).
(DOCX)

S3 Table The intracellular ROS level in LoVo (panel a), MCF7 (panel b), and MCF-10A (panel c) after incubation (1 h at 37°C) with the PRBC supernatants (5%) in the absence or presence of cisPt (panel a and b: 25 μM; panel c: 40 μM).
(DOCX)

S4 Table The DNA damage, measured as a percentage of tail DNA in the alkaline comet assay, in LoVo, MCF7, and MCF-10A cells pre-incubated for 1 h at 37°C with the PRBC supernatants (5%). (DOCX)
