# Supplementary material for: Responses of human colon and breast adenocarcinoma cell lines (LoVo, MCF7) and non-tumorigenic mammary epithelial cells (MCF-10A) to the acellular fraction of packed red blood cells in the presence and absence of cisplatin
Source: PLoS One. 2022 Jul 8;17(7):e0271193. doi: 10.1371/journal.pone.0271193 (PMC9269965; doi:10.1371/journal.pone.0271193)
Supplement: S1 Table — Viability of LoVo (panel a), MCF7 (panel b), and MCF-10A (panel c) cells after incubation (24 h) with the PRBC supernatants (5%) in the absence or presence of cisPt (panel a and b: 25 μM; panel c: 40 μM) evaluated using the CCK-8 assay. (DOCX) [file pone.0271193.s001.docx]

**S1 Table. Viability of LoVo (panel a), MCF7 (panel b), and MCF-10A (panel c) cells after incubation (24 h) with the PRBC supernatants (5%) in the absence or presence of cisPt (panel a and b: 25 µM; panel c: 40 µM) evaluated using the CCK-8 assay.**

| **LoVo (panel a)** | | | | | | | | | | | | | | | |
| --- | --- | --- | --- | --- | --- | --- | --- | --- | --- | --- | --- | --- | --- | --- | --- |
| **Control** | 100,0 | 100,0 | 100,0 | 100,0 | 100,0 | 100,0 | 100,0 | 100,0 | 100,0 | 100,0 | 100,0 | 100,0 | 100,0 | 100,0 | 100,0 |
| **sNLR1** | 87,4 | 85,4 | 84,0 | 74,9 | 82,9 | 85,3 | 89,6 | 82,9 | 79,1 | 72,0 | 81,4 | 89,3 | 76,4 | 90,1 | 82,4 |
| **sNLR42** | 107,1 | 98,5 | 96,5 | 76,5 | 105,9 | 95,4 | 101,5 | 91,2 | 79,5 | 109,1 | 85,3 | 71,0 | 94,6 | 99,5 | 105,6 |
| **sLR1** | 96,0 | 78,1 | 79,8 | 62,2 | 95,2 | 86,5 | 78,2 | 61,3 | 59,9 | 95,6 | 79,7 | 69,8 | 79,8 | 82,6 | 79,9 |
| **sLR42** | 97,1 | 76,1 | 71,0 | 86,6 | 86,8 | 85,7 | 79,8 | 82,3 | 63,6 | 81,6 | 92,0 | 95,8 | 107,3 | 99,6 | 86,1 |
| **LoVo + cisPt (panel a)** | | | | | | | | | | | | | | | |
| **Control** | 79,3 | 54,3 | 77,0 | 72,7 | 59,8 | 78,6 | 61,5 | 69,4 | 70,9 | 78,5 | 72,9 | 81,5 | 62,5 | 57,9 | 85,6 |
| **sNLR1** | 48,1 | 59,4 | 44,6 | 52,2 | 69,5 | 51,2 | 47,2 | 39,9 | 47,6 | 51,7 | 59,8 | 52,6 | 33,5 | 58,6 | 51,1 |
| **sNLR42** | 84,8 | 71,2 | 66,8 | 72,4 | 78,9 | 86,3 | 79,2 | 56,2 | 76,5 | 71,2 | 73,8 | 65,3 | 72,3 | 73,5 | 78,6 |
| **sLR1** | 79,9 | 77,8 | 57,5 | 65,2 | 69,8 | 78,5 | 57,0 | 69,4 | 85,2 | 73,6 | 71,6 | 80,3 | 62,3 | 71,0 | 52,5 |
| **sLR42** | 77,2 | 78,8 | 48,0 | 58,6 | 59,2 | 59,0 | 50,3 | 67,3 | 73,4 | 48,2 | 52,3 | 62,0 | 67,3 | 63,4 | 61,8 |
| **MCF7 (panel b)** | | | | | | | | | | | | | | | |
| **Control** | 100,0 | 100,0 | 100,0 | 100,0 | 100,0 | 100,0 | 100,0 | 100,0 | 100,0 | 100,0 | 100,0 | 100,0 | 100,0 | 100,0 | 100,0 |
| **sNLR1** | 80,2 | 73,4 | 76,9 | 85,9 | 80,7 | 69,5 | 69,4 | 76,8 | 78,9 | 82,6 | 74,2 | 71,3 | 76,8 | 79,8 | 74,9 |
| **sNLR42** | 90,8 | 85,1 | 91,5 | 81,5 | 92,6 | 76,5 | 82,6 | 88,1 | 91,5 | 85,6 | 88,1 | 89,6 | 98,5 | 86,3 | 91,5 |
| **sLR1** | 65,4 | 77,6 | 74,0 | 73,6 | 78,5 | 80,6 | 81,5 | 72,6 | 71,2 | 70,2 | 72,3 | 68,5 | 67,5 | 64,5 | 65,9 |
| **sLR42** | 95,2 | 72,8 | 84,0 | 84,0 | 85,9 | 87,4 | 92,5 | 98,5 | 84,2 | 99,8 | 72,1 | 69,8 | 62,4 | 84,0 | 86,9 |
| **MCF7 + cisPt (panel b)** | | | | | | | | | | | | | | | |
| **Control** | 76,1 | 73,4 | 75,8 | 74,7 | 75,4 | 71,5 | 74,8 | 74,5 | 79,8 | 75,4 | 71,4 | 76,5 | 74,7 | 75,9 | 71,0 |
| **sNLR1** | 95,0 | 61,5 | 68,0 | 76,8 | 71,0 | 70,0 | 79,8 | 89,8 | 73,7 | 63,1 | 74,5 | 68,4 | 74,8 | 71,2 | 67,5 |
| **sNLR42** | 76,7 | 81,6 | 79,0 | 78,1 | 82,6 | 79,1 | 79,5 | 79,4 | 89,5 | 74,8 | 79,8 | 75,2 | 71,4 | 82,5 | 79,4 |
| **sLR1** | 77,5 | 56,2 | 71,0 | 78,5 | 87,8 | 61,2 | 76,5 | 61,0 | 74,2 | 67,8 | 65,2 | 62,3 | 60,5 | 64,5 | 59,2 |
| **sLR42** | 80,1 | 61,9 | 76,0 | 72,6 | 69,8 | 87,6 | 84,3 | 98,2 | 78,5 | 74,2 | 69,7 | 64,5 | 53,6 | 51,2 | 68,1 |
| **MCF-10A (panel c)** | | | | | | | | | | | | | | | |
| **Control** | 100,0 | 100,0 | 100,0 | 100,0 | 100,0 | 100,0 | 100,0 | 100,0 | 100,0 | 100,0 | 100,0 | 100,0 | 100,0 | 100,0 | 100,0 |
| **sNLR1** | 110,3 | 99,0 | 112,0 | 112,6 | 126,3 | 103,6 | 101,6 | 99,8 | 98,5 | 99,1 | 99,8 | 109,8 | 107,1 | 118,6 | 108,6 |
| **sNLR42** | 120,5 | 115,0 | 110,0 | 120,0 | 130,0 | 99,0 | 129,8 | 99,7 | 126,9 | 137,2 | 99,7 | 116,5 | 110,3 | 110,3 | 112,6 |
| **sLR1** | 110,2 | 97,0 | 115,0 | 109,8 | 109,7 | 120,9 | 101,6 | 99,8 | 110,6 | 108,5 | 99,8 | 99,8 | 105,6 | 112,6 | 109,7 |
| **sLR42** | 125,8 | 105,0 | 115,0 | 125,0 | 120,5 | 132,0 | 115,6 | 115,6 | 125,6 | 106,3 | 119,8 | 117,6 | 109,6 | 107,6 | 124,6 |
| **MCF-10A + cisPt (panel c)** | | | | | | | | | | | | | | | |
| **Control** | 68,8 | 75,0 | 70,0 | 60,0 | 68,9 | 79,5 | 74,5 | 62,5 | 60,2 | 60,8 | 67,3 | 68,2 | 68,7 | 68,2 | 72,9 |
| **sNLR1** | 104,2 | 91,0 | 94,0 | 90,0 | 95,8 | 92,3 | 90,2 | 92,5 | 95,2 | 99,8 | 109,4 | 84,3 | 86,8 | 94,8 | 102,3 |
| **sNLR42** | 104,5 | 86,0 | 110,0 | 106,8 | 98,6 | 82,6 | 92,1 | 106,2 | 115,3 | 79,6 | 102,3 | 108,6 | 105,9 | 106,9 | 97,2 |
| **sLR1** | 86,9 | 96,1 | 101,0 | 106,5 | 92,6 | 90,5 | 99,8 | 93,5 | 91,0 | 82,6 | 86,9 | 96,8 | 102,3 | 99,7 | 94,7 |
| **sLR42** | 98,0 | 102,0 | 112,0 | 107,0 | 115,0 | 109,8 | 99,7 | 109,5 | 97,8 | 108,9 | 116,5 | 99,3 | 109,8 | 115,6 | 101,2 |
