# Supplementary material for: Responses of human colon and breast adenocarcinoma cell lines (LoVo, MCF7) and non-tumorigenic mammary epithelial cells (MCF-10A) to the acellular fraction of packed red blood cells in the presence and absence of cisplatin
Source: PLoS One. 2022 Jul 8;17(7):e0271193. doi: 10.1371/journal.pone.0271193 (PMC9269965; doi:10.1371/journal.pone.0271193)
Supplement: S2 Table — Proliferation of LoVo (panel a), MCF7 (panel b), and MCF-10A (panel c) cells, assessed by the BrdU incorporation during DNA synthesis, after incubation (24 h) with the PRBC supernatants (5%) in the absence or presence of cisPt (panel a and b: 25 μM; panel c: 40 μM). (DOCX) [file pone.0271193.s002.docx]

**S2 Table. Proliferation of LoVo (panel a), MCF7 (panel b), and MCF-10A (panel c) cells, assessed by the BrdU incorporation during DNA synthesis, after incubation (24 h) with the PRBC supernatants (5%) in the absence or presence of cisPt (panel a and b: 25 µM; panel c: 40 µM).**

| **LoVo (panel a)** | | | | | | | | | | | | | | | |
| --- | --- | --- | --- | --- | --- | --- | --- | --- | --- | --- | --- | --- | --- | --- | --- |
| **Control** | 100,0 | 100,0 | 100,0 | 100,0 | 100,0 | 100,0 | 100,0 | 100,0 | 100,0 | 100,0 | 100,0 | 100,0 | 100,0 | 100,0 | 100,0 |
| **sNLR1** | 71,0 | 75,2 | 62,0 | 59,0 | 69,2 | 51,9 | 71,3 | 65,3 | 67,2 | 67,6 | 65,3 | 69,8 | 71,2 | 69,7 | 66,8 |
| **sNLR42** | 86,0 | 91,0 | 95,0 | 91,3 | 90,7 | 98,6 | 84,3 | 79,6 | 83,6 | 98,6 | 94,5 | 98,6 | 85,6 | 90,3 | 92,3 |
| **sLR1** | 92,0 | 87,6 | 68,0 | 59,0 | 98,0 | 81,2 | 82,6 | 98,7 | 53,6 | 81,6 | 92,6 | 95,6 | 81,2 | 62,3 | 79,0 |
| **sLR42** | 87,0 | 82,6 | 97,0 | 88,9 | 87,6 | 99,8 | 90,6 | 79,6 | 89,6 | 88,2 | 87,2 | 98,5 | 87,5 | 79,8 | 88,9 |
| **LoVo + cisPt (panel a)** | | | | | | | | | | | | | | | |
| **Control** | 49,6 | 59,0 | 45,0 | 50,0 | 53,6 | 48,5 | 52,1 | 48,6 | 47,5 | 46,5 | 60,2 | 46,8 | 40,6 | 53,9 | 61,5 |
| **sNLR1** | 53,9 | 58,0 | 48,0 | 72,0 | 68,9 | 57,6 | 70,6 | 56,9 | 50,2 | 62,3 | 62,5 | 59,8 | 61,3 | 45,9 | 42,3 |
| **sNLR42** | 63,3 | 76,0 | 70,0 | 63,9 | 78,6 | 57,6 | 75,6 | 75,6 | 64,8 | 61,9 | 62,3 | 69,2 | 69,8 | 78,6 | 79,8 |
| **sLR1** | 56,8 | 49,0 | 48,0 | 61,0 | 56,9 | 68,9 | 61,5 | 53,6 | 49,1 | 49,7 | 49,8 | 46,5 | 58,9 | 50,3 | 45,3 |
| **sLR42** | 60,2 | 65,0 | 55,0 | 69,5 | 73,0 | 52,3 | 70,6 | 69,3 | 62,3 | 59,2 | 50,3 | 50,9 | 49,2 | 49,2 | 65,3 |
| **MCF7 (panel b)** | | | | | | | | | | | | | | | |
| **Control** | 100,0 | 100,0 | 100,0 | 100,0 | 100,0 | 100,0 | 100,0 | 100,0 | 100,0 | 100,0 | 100,0 | 100,0 | 100,0 | 100,0 | 100,0 |
| **sNLR1** | 85,8 | 90,2 | 79,4 | 89,2 | 84,1 | 99,8 | 85,6 | 85,6 | 80,6 | 78,9 | 68,9 | 84,5 | 85,9 | 88,7 | 89,8 |
| **sNLR42** | 90,3 | 97,6 | 105,4 | 97,8 | 102,5 | 99,8 | 97,8 | 106,9 | 95,8 | 92,6 | 99,7 | 88,9 | 89,8 | 102,5 | 99,8 |
| **sLR1** | 75,0 | 85,0 | 82,0 | 78,9 | 75,6 | 89,5 | 72,6 | 89,6 | 91,5 | 85,6 | 72,3 | 80,3 | 75,6 | 74,6 | 81,6 |
| **sLR42** | 93,3 | 91,3 | 87,0 | 98,5 | 95,2 | 88,9 | 90,5 | 87,9 | 90,5 | 88,9 | 87,6 | 90,5 | 87,7 | 90,5 | 89,9 |
| **MCF7 + cisPt (panel b)** | | | | | | | | | | | | | | | |
| **Control** | 51,2 | 42,0 | 56,0 | 43,0 | 52,3 | 48,9 | 56,3 | 49,5 | 41,0 | 42,3 | 52,3 | 48,1 | 52,3 | 41,3 | 44,6 |
| **sNLR1** | 25,8 | 32,6 | 39,3 | 21,0 | 36,5 | 29,9 | 30,2 | 31,6 | 25,6 | 27,3 | 20,6 | 36,5 | 39,8 | 21,3 | 26,9 |
| **sNLR42** | 37,5 | 46,9 | 34,7 | 39,2 | 37,8 | 46,9 | 33,5 | 37,6 | 42,9 | 39,4 | 39,7 | 42,3 | 49,6 | 32,6 | 34,0 |
| **sLR1** | 38,2 | 48,7 | 45,3 | 44,3 | 38,6 | 44,2 | 48,9 | 36,8 | 34,6 | 49,8 | 39,8 | 49,9 | 51,3 | 44,9 | 44,6 |
| **sLR42** | 44,7 | 52,0 | 39,4 | 43,7 | 52,6 | 44,2 | 40,3 | 48,6 | 45,8 | 40,8 | 52,6 | 44,0 | 42,6 | 39,8 | 42,6 |
| **MCF-10A (panel c)** | | | | | | | | | | | | | | | |
| **Control** | 100,0 | 100,0 | 100,0 | 100,0 | 100,0 | 100,0 | 100,0 | 100,0 | 100,0 | 100,0 | 100,0 | 100,0 | 100,0 | 100,0 | 100,0 |
| **sNLR1** | 124,2 | 119,0 | 121,0 | 99,0 | 119,1 | 120,5 | 99,9 | 114,3 | 122,6 | 128,3 | 99,8 | 123,5 | 120,3 | 110,6 | 115,2 |
| **sNLR42** | 136,0 | 142,7 | 130,7 | 129,7 | 135,6 | 140,6 | 130,2 | 128,9 | 130,8 | 125,6 | 132,5 | 133,5 | 150,3 | 136,5 | 136,5 |
| **sLR1** | 95,8 | 102,4 | 93,6 | 86,0 | 95,6 | 99,8 | 95,4 | 88,9 | 92,5 | 88,9 | 98,6 | 103,6 | 99,8 | 81,3 | 94,2 |
| **sLR42** | 126,8 | 115,0 | 135,2 | 110,6 | 136,5 | 129,7 | 139,8 | 129,3 | 119,6 | 120,6 | 120,6 | 120,5 | 127,8 | 135,0 | 118,6 |
| **MCF-10A + cisPt (panel c)** | | | | | | | | | | | | | | | |
| **Control** | 38,5 | 48,0 | 33,0 | 37,0 | 38,4 | 45,9 | 33,5 | 30,2 | 36,5 | 49,7 | 35,9 | 47,9 | 38,4 | 39,8 | 34,5 |
| **sNLR1** | 43,1 | 53,0 | 59,0 | 42,0 | 52,6 | 48,9 | 59,8 | 52,3 | 42,3 | 49,7 | 52,6 | 47,8 | 40,0 | 49,5 | 46,5 |
| **sNLR42** | 55,3 | 65,0 | 59,0 | 53,0 | 55,8 | 55,7 | 69,8 | 65,2 | 62,5 | 60,3 | 57,6 | 52,3 | 51,1 | 56,5 | 52,0 |
| **sLR1** | 30,3 | 43,0 | 30,5 | 35,0 | 41,3 | 36,5 | 45,6 | 34,9 | 42,6 | 29,8 | 37,6 | 21,2 | 27,9 | 42,8 | 49,8 |
| **sLR42** | 44,1 | 68,0 | 85,0 | 50,0 | 69,8 | 52,3 | 39,2 | 69,7 | 44,6 | 80,3 | 46,5 | 63,5 | 87,6 | 56,2 | 69,9 |
