# Supplementary material for: Responses of human colon and breast adenocarcinoma cell lines (LoVo, MCF7) and non-tumorigenic mammary epithelial cells (MCF-10A) to the acellular fraction of packed red blood cells in the presence and absence of cisplatin
Source: PLoS One. 2022 Jul 8;17(7):e0271193. doi: 10.1371/journal.pone.0271193 (PMC9269965; doi:10.1371/journal.pone.0271193)
Supplement: S3 Table — The intracellular ROS level in LoVo (panel a), MCF7 (panel b), and MCF-10A (panel c) after incubation (1 h at 37°C) with the PRBC supernatants (5%) in the absence or presence of cisPt (panel a and b: 25 μM; panel c: 40 μM). (DOCX) [file pone.0271193.s003.docx]

**S3 Table. The intracellular ROS level in LoVo (panel a), MCF7 (panel b), and MCF-10A (panel c) after incubation (1 h at 37ºC) with the PRBC supernatants (5%) in the absence or presence of cisPt (panel a and b: 25 µM; panel c: 40 µM).**

| **LoVo (panel a)** | | | | | **LoVo + cisPt (panel a)** | | | | |
| --- | --- | --- | --- | --- | --- | --- | --- | --- | --- |
| **Control** | **sNLR1** | **sNLR42** | **sLR1** | **sLR42** | **Control** | **sNLR1** | **sNLR42** | **sLR1** | **sLR42** |
| 100,0 | 115,8 | 139,0 | 136,5 | 136,5 | 128,0 | 117,3 | 188,5 | 200,1 | 176,7 |
| 100,0 | 112,5 | 147,5 | 122,0 | 131,8 | 135,0 | 132,1 | 171,0 | 180,0 | 164,0 |
| 100,0 | 119,1 | 130,6 | 130,1 | 141,3 | 120,3 | 102,5 | 150,0 | 140,0 | 190,0 |
| 100,0 | 99,0 | 178,0 | 120,0 | 166,4 | 128,5 | 109,5 | 250,0 | 205,6 | 178,5 |
| 100,0 | 118,5 | 173,8 | 125,6 | 115,0 | 135,2 | 108,6 | 243,5 | 158,6 | 198,5 |
| 100,0 | 98,2 | 183,8 | 135,0 | 110,5 | 124,5 | 134,2 | 195,4 | 142,5 | 189,5 |
| 100,0 | 117,2 | 193,8 | 136,8 | 126,5 | 127,5 | 109,5 | 168,5 | 198,5 | 187,5 |
| 100,0 | 107,9 | 198,2 | 134,6 | 138,0 | 139,5 | 118,2 | 154,8 | 175,0 | 192,5 |
| 100,0 | 120,3 | 161,2 | 132,4 | 162,3 | 130,5 | 132,5 | 198,5 | 148,5 | 165,8 |
| 100,0 | 95,3 | 139,0 | 139,5 | 138,5 | 128,6 | 127,4 | 225,3 | 200,9 | 179,5 |
| 100,0 | 105,2 | 147,5 | 124,3 | 139,5 | 124,5 | 132,5 | 192,0 | 152,6 | 184,5 |
| 100,0 | 115,3 | 136,5 | 121,8 | 120,5 | 129,2 | 104,6 | 195,5 | 158,5 | 153,5 |
| 100,0 | 121,3 | 110,6 | 126,8 | 129,5 | 131,2 | 105,9 | 168,5 | 201,3 | 186,4 |
| 100,0 | 111,1 | 130,1 | 131,2 | 156,8 | 134,5 | 105,0 | 175,2 | 186,2 | 175,6 |
| 100,0 | 118,5 | 130,2 | 129,8 | 169,2 | 127,8 | 117,5 | 198,5 | 178,9 | 179,5 |
| 100,0 | 113,1 | 141,0 | 124,6 | 149,2 | 122,3 | 116,2 | 206,3 | 158,0 | 164,5 |
| 100,0 | 116,3 | 162,3 | 127,8 | 125,8 | 130,2 | 115,2 | 178,2 | 147,5 | 186,5 |
| 100,0 | 112,3 | 139,0 | 129,5 | 139,5 | 138,5 | 107,2 | 200,5 | 210,5 | 170,5 |
| 100,0 | 98,0 | 147,5 | 119,5 | 146,5 | 129,4 | 103,5 | 187,5 | 149,5 | 147,5 |
| 100,0 | 118,6 | 132,5 | 132,5 | 129,5 | 120,5 | 125,3 | 174,5 | 182,3 | 185,6 |
| 100,0 | 96,2 | 158,6 | 137,0 | 145,2 | 127,5 | 135,2 | 198,5 | 198,5 | 185,5 |
| 100,0 | 113,5 | 130,5 | 119,2 | 168,8 | 139,5 | 117,5 | 162,0 | 165,2 | 178,6 |
| 100,0 | 119,2 | 142,3 | 119,8 | 125,6 | 120,2 | 103,5 | 185,6 | 152,3 | 185,6 |
| 100,0 | 115,6 | 134,5 | 111,3 | 121,3 | 115,3 | 128,5 | 189,8 | 173,5 | 164,4 |
| 100,0 | 112,3 | 132,0 | 109,8 | 120,3 | 107,9 | 123,1 | 188,6 | 169,5 | 152,6 |
| **MCF7 (panel b)** | | | | | **MCF7 + cisPt (panel b)** | | | | |
| **Control** | **sNLR1** | **sNLR42** | **sLR1** | **sLR42** | **Control** | **sNLR1** | **sNLR42** | **sLR1** | **sLR42** |
| 100,0 | 122,1 | 172,0 | 132,1 | 150,5 | 199,0 | 196,1 | 232,2 | 300,7 | 279,6 |
| 100,0 | 135,3 | 189,0 | 120,3 | 140,3 | 180,7 | 225,0 | 185,0 | 250,1 | 190,0 |
| 100,0 | 129,0 | 165,7 | 129,7 | 168,4 | 190,6 | 176,7 | 198,5 | 261,0 | 250,0 |
| 100,0 | 110,0 | 100,0 | 160,0 | 189,4 | 165,0 | 198,5 | 185,5 | 220,0 | 198,5 |
| 100,0 | 125,6 | 180,0 | 124,3 | 90,3 | 220,0 | 185,8 | 195,5 | 150,0 | 205,6 |
| 100,0 | 127,5 | 165,8 | 128,5 | 174,1 | 225,2 | 214,2 | 245,5 | 302,2 | 285,5 |
| 100,0 | 122,2 | 169,5 | 132,5 | 189,8 | 189,5 | 206,2 | 223,5 | 269,5 | 225,6 |
| 100,0 | 135,9 | 189,5 | 132,2 | 152,2 | 189,5 | 208,5 | 248,5 | 264,5 | 198,5 |
| 100,0 | 132,4 | 182,3 | 127,4 | 175,6 | 196,7 | 199,5 | 195,5 | 295,0 | 185,6 |
| 100,0 | 108,5 | 187,2 | 129,9 | 126,5 | 226,5 | 207,5 | 196,6 | 300,6 | 226,5 |
| 100,0 | 126,6 | 106,5 | 135,6 | 168,2 | 196,5 | 187,5 | 236,3 | 168,5 | 244,2 |
| 100,0 | 138,5 | 109,5 | 132,0 | 158,6 | 175,2 | 178,9 | 198,5 | 179,5 | 225,5 |
| 100,0 | 132,5 | 172,2 | 142,3 | 169,8 | 185,2 | 206,2 | 185,5 | 165,5 | 225,5 |
| 100,0 | 117,0 | 153,6 | 128,5 | 182,5 | 163,2 | 176,5 | 187,2 | 200,3 | 256,5 |
| 100,0 | 128,6 | 165,3 | 120,3 | 98,2 | 165,5 | 202,3 | 236,5 | 236,5 | 265,5 |
| 100,0 | 135,5 | 182,3 | 124,5 | 92,5 | 182,3 | 189,9 | 184,2 | 305,2 | 278,5 |
| 100,0 | 127,5 | 184,6 | 132,0 | 145,2 | 184,2 | 200,5 | 198,5 | 152,2 | 245,5 |
| 100,0 | 124,4 | 189,2 | 140,2 | 198,2 | 198,5 | 204,5 | 214,3 | 235,0 | 225,6 |
| 100,0 | 116,5 | 172,2 | 159,5 | 152,3 | 199,2 | 196,2 | 205,3 | 258,2 | 256,5 |
| 100,0 | 99,3 | 156,6 | 148,5 | 145,5 | 204,5 | 192,5 | 198,5 | 256,3 | 205,6 |
| 100,0 | 126,9 | 163,3 | 169,6 | 98,2 | 189,5 | 178,5 | 175,8 | 223,3 | 224,5 |
| 100,0 | 124,5 | 152,3 | 136,2 | 102,2 | 190,3 | 185,0 | 198,5 | 245,2 | 298,5 |
| 100,0 | 136,5 | 142,3 | 125,2 | 136,5 | 175,2 | 209,5 | 198,2 | 234,5 | 278,5 |
| 100,0 | 110,2 | 113,2 | 139,2 | 142,3 | 192,2 | 225,6 | 235,4 | 215,3 | 265,5 |
| 100,0 | 109,5 | 169,2 | 136,5 | 145,2 | 189,9 | 231,2 | 256,6 | 220,2 | 256,6 |
| **MCF-10A (panel c)** | | | | | **MCF-10A + cisPt (panel c)** | | | | |
| **Control** | **sNLR1** | **sNLR42** | **sLR1** | **sLR42** | **Control** | **sNLR1** | **sNLR42** | **sLR1** | **sLR42** |
| 100,0 | 72,3 | 87,4 | 93,0 | 104,6 | 131,5 | 121,0 | 153,0 | 104,3 | 111,1 |
| 100,0 | 75,7 | 85,7 | 95,0 | 95,3 | 140,4 | 142,0 | 206,9 | 120,5 | 133,8 |
| 100,0 | 69,7 | 89,3 | 82,7 | 110,6 | 135,0 | 125,5 | 156,0 | 127,0 | 122,0 |
| 100,0 | 89,0 | 95,0 | 96,2 | 90,6 | 147,0 | 129,5 | 205,2 | 125,3 | 166,0 |
| 100,0 | 72,5 | 89,5 | 82,6 | 95,5 | 145,5 | 148,5 | 152,3 | 112,2 | 112,5 |
| 100,0 | 78,5 | 87,5 | 84,2 | 98,4 | 147,8 | 129,8 | 256,2 | 125,6 | 152,5 |
| 100,0 | 69,5 | 89,6 | 95,3 | 90,3 | 148,5 | 124,5 | 198,2 | 117,6 | 168,2 |
| 100,0 | 85,5 | 85,9 | 81,2 | 110,6 | 140,3 | 126,4 | 175,2 | 113,2 | 119,8 |
| 100,0 | 85,6 | 89,8 | 86,5 | 108,7 | 146,8 | 127,2 | 169,5 | 106,5 | 126,5 |
| 100,0 | 86,5 | 82,3 | 94,2 | 96,5 | 136,5 | 135,6 | 198,5 | 109,5 | 145,2 |
| 100,0 | 72,5 | 80,2 | 95,2 | 119,5 | 130,3 | 142,3 | 145,2 | 116,2 | 148,8 |
| 100,0 | 71,2 | 81,3 | 98,5 | 97,5 | 140,2 | 142,3 | 132,5 | 114,1 | 136,5 |
| 100,0 | 85,5 | 89,5 | 91,2 | 85,6 | 143,2 | 124,5 | 163,2 | 127,5 | 152,6 |
| 100,0 | 72,3 | 84,6 | 82,3 | 94,2 | 132,5 | 129,8 | 179,2 | 123,2 | 147,0 |
| 100,0 | 70,2 | 95,6 | 87,2 | 93,2 | 139,0 | 139,5 | 165,5 | 116,5 | 156,5 |
| 100,0 | 82,3 | 92,3 | 93,6 | 104,2 | 130,2 | 127,4 | 152,3 | 103,2 | 160,2 |
| 100,0 | 65,5 | 87,1 | 98,3 | 96,2 | 137,5 | 129,0 | 142,3 | 127,4 | 124,5 |
| 100,0 | 69,8 | 95,6 | 96,3 | 90,3 | 134,5 | 134,2 | 145,2 | 119,5 | 154,5 |
| 100,0 | 72,6 | 96,5 | 96,5 | 103,2 | 134,2 | 149,8 | 142,0 | 127,7 | 125,5 |
| 100,0 | 68,5 | 84,5 | 84,5 | 108,5 | 130,2 | 139,5 | 148,0 | 116,2 | 136,5 |
| 100,0 | 71,2 | 82,4 | 86,5 | 96,5 | 146,2 | 124,8 | 145,2 | 119,2 | 103,2 |
| 100,0 | 86,5 | 93,2 | 84,8 | 97,9 | 149,8 | 129,8 | 198,3 | 125,5 | 120,5 |
| 100,0 | 63,2 | 91,2 | 91,2 | 109,5 | 130,2 | 141,2 | 187,3 | 113,5 | 99,8 |
| 100,0 | 93,2 | 98,5 | 86,3 | 116,5 | 137,5 | 112,3 | 198,2 | 106,2 | 105,5 |
| 100,0 | 87,5 | 98,5 | 92,5 | 92,5 | 127,8 | 110,3 | 185,2 | 112,2 | 101,2 |
