# Supplementary material for: Responses of human colon and breast adenocarcinoma cell lines (LoVo, MCF7) and non-tumorigenic mammary epithelial cells (MCF-10A) to the acellular fraction of packed red blood cells in the presence and absence of cisplatin
Source: PLoS One. 2022 Jul 8;17(7):e0271193. doi: 10.1371/journal.pone.0271193 (PMC9269965; doi:10.1371/journal.pone.0271193)
Supplement: S4 Table — (DOCX) [file pone.0271193.s004.docx]

**S4 Table. The DNA damage, measured as a percentage of tail DNA in the alkaline comet assay, in LoVo, MCF7, and MCF-10A cells pre-incubated for 1 h at 37ºC with the PRBC supernatants (5%).**

| **LoVo** | | | | | | | | | | | | | | | |
| --- | --- | --- | --- | --- | --- | --- | --- | --- | --- | --- | --- | --- | --- | --- | --- |
| **Control** | 1,7 | 1,2 | 2,0 | 2,0 | 2,6 | 3,5 | 1,9 | 1,6 | 1,2 | 1,9 | 1,3 | 1,8 | 2,5 | 1,3 | 1,9 |
| **sNLR1** | 2,5 | 2,2 | 2,2 | 3,1 | 1,2 | 2,9 | 3,0 | 1,7 | 1,8 | 1,3 | 1,2 | 1,9 | 2,0 | 2,9 | 3,1 |
| **sNLR42** | 3,0 | 3,6 | 1,6 | 1,3 | 1,3 | 1,4 | 2,8 | 3,9 | 1,9 | 1,5 | 1,7 | 1,5 | 2,2 | 2,6 | 2,5 |
| **sLR1** | 2,5 | 2,6 | 3,6 | 3,7 | 2,1 | 2,3 | 3,7 | 2,5 | 2,9 | 2,1 | 3,5 | 2,1 | 3,5 | 3,4 | 3,6 |
| **sLR42** | 2,6 | 1,6 | 2,7 | 3,0 | 1,7 | 2,3 | 2,9 | 1,6 | 2,3 | 2,4 | 2,9 | 3,1 | 1,6 | 1,2 | 2,3 |
| **MCF7** | | | | | | | | | | | | | | | |
| **Control** | 1,7 | 1,2 | 1,5 | 2,0 | 1,9 | 1,1 | 1,0 | 1,5 | 1,6 | 1,8 | 1,9 | 1,2 | 1,1 | 1,0 | 1,5 |
| **sNLR1** | 3,1 | 3,4 | 2,8 | 1,9 | 2,8 | 3,9 | 2,1 | 2,8 | 2,6 | 1,8 | 3,3 | 2,8 | 2,7 | 2,8 | 2,8 |
| **sNLR42** | 1,3 | 2,4 | 2,0 | 2,2 | 1,8 | 1,1 | 1,0 | 1,8 | 1,6 | 2,6 | 2,0 | 2,2 | 2,9 | 1,0 | 1,8 |
| **sLR1** | 4,1 | 3,8 | 3,3 | 4,2 | 3,8 | 4,6 | 4,0 | 3,1 | 3,0 | 3,5 | 3,7 | 3,5 | 3,3 | 3,5 | 3,9 |
| **sLR42** | 2,1 | 4,6 | 3,4 | 2,0 | 4,9 | 3,5 | 3,4 | 4,0 | 3,9 | 3,0 | 2,1 | 3,4 | 2,1 | 4,6 | 4,7 |
| **MCF-10A** | | | | | | | | | | | | | | | |
| **Control** | 2,7 | 2,2 | 1,6 | 2,7 | 2,8 | 2,1 | 1,6 | 2,9 | 2,3 | 2,4 | 2,9 | 2,0 | 1,9 | 1,8 | 2,3 |
| **sNLR1** | 2,9 | 2,0 | 2,5 | 2,0 | 1,5 | 3,1 | 2,9 | 3,6 | 2,2 | 2,5 | 1,8 | 2,5 | 2,7 | 2,5 | 3,0 |
| **sNLR42** | 3,0 | 2,0 | 1,7 | 2,2 | 3,1 | 2,4 | 2,0 | 1,6 | 3,2 | 2,2 | 2,5 | 1,0 | 1,8 | 2,6 | 2,4 |
| **sLR1** | 2,9 | 2,1 | 2,9 | 2,5 | 1,9 | 2,5 | 3,1 | 3,9 | 1,2 | 2,5 | 1,8 | 1,7 | 2,7 | 2,9 | 2,5 |
| **sLR42** | 3,1 | 2,7 | 1,6 | 1,2 | 2,2 | 2,2 | 3,0 | 1,9 | 1,2 | 2,4 | 2,2 | 3,5 | 3,9 | 2,2 | 1,6 |
